# The Work of the Late F. W. Pavy

**Published:** 1911-09-30

**Authors:** 


### The Work of the late F. W. Pavy.

In his eighty-third year Dr. Frederick William Pavy died on September 19, after a short illness. His reputation on questions of metabolism was world-wide, and his exhaustive researches on diabetes form the basis of much of our present knowledge on this subject. For many years he had been working in his own private laboratory on the chemistry of carbohydrate digestion and pathological sugar formation, assisted by Dr. Hubert Bywaters, D.Sc. During the whole of his long career Dr. Pavy has been associated with Guy's Hospital, for it was at this school that he received his medical education and mounted the successive steps leading from Assistant Physician to Consulting Physician. Soon after taking the M.D. (London) in 1853, he was appointed Lecturer on Physiology, Zoology, and Comparative Anatomy. In 1860 Dr. Pavy was elected a Fellow of the Royal College of Physicians, in which body he filled the various offices held by its most distinguished members. He was in turn Goulstonian Lecturer, Croonian Lecturer, Censor and Harveian Orator, and received the Baly Gold Medal in 1901. He was President of the Royal Medical and Chirurgical Society and of the Pathological Society, besides being a member of many other learned societies at home and abroad. In 1909 he received the Prix Godard of the Paris Academie de Médecine. When he became a full physician to Guy's Hospital he took up the Lectureship on the Principles and Practice of Medicine to the great and lasting advantage of the students of that hospital. Dr. Pavy's very numerous contributions to the literature on diabetes and carbohydrate metabolism speak for themselves, while the widespread expressions of admiration with which his eightieth birthday was honoured by the medical profession all over Europe are witness of the high esteem in which he and his work were held.

